# The Role of Adenosine in Alzheimer’s Disease

**DOI:** 10.2174/157015909789152119

**Published:** 2009-09

**Authors:** Anisur Rahman

**Affiliations:** Legacy Research, R.S Dow Neurobiology Laboratories, 1225 NE 2nd Avenue, Portland OR 97232, USA

**Keywords:** Adenosine receptor, Alzheimer’s disease, amyloid beta, caffeine, cognition, neuromodulation.

## Abstract

Alzheimer’s disease (AD) is a neurodegenerative disorder of the central nervous system manifested by cognitive and memory deterioration, a variety of neuropsychiatric symptoms, behavioral disturbances, and progressive impairment of daily life activities. Current pharmacotherapies are restricted to symptomatic interventions but do not prevent progressive neuronal degeneration. Therefore, new therapeutic strategies are needed to intervene with these progressive pathological processes. In the past several years adenosine, a ubiquitously released purine ribonucleoside, has become important for its neuromodulating capability and its emerging positive experimental effects in neurodegenerative diseases. Recent research suggests that adenosine receptors play important roles in the modulation of cognitive function. The present paper attempts to review published reports and data from different studies showing the evidence of a relationship between adenosinergic function and AD-related cognitive deficits. Epidemiological studies have found an association between coffee (a nonselective adenosine receptor antagonist) consumption and improved cognitive function in AD patients and in the elderly. Long-term administration of caffeine in transgenic animal models showed a reduced amyloid burden in brain with better cognitive performance. Antagonists of adenosine A2A receptors mimic these beneficial effects of caffeine on cognitive function. Neuronal cell cultures with amyloid beta in the presence of an A2A receptor antagonist completely prevented amyloid beta-induced neurotoxicity. These findings suggest that the adenosinergic system constitutes a new therapeutic target for AD, and caffeine and A2A receptor antagonists may have promise to manage cognitive dysfunction in AD.

## INTRODUCTION

Alzheimer’s disease (AD) is a progressive neurodegenerative disease associated with the most common form of dementia in the elderly and accounting for around 50-60% of dementia in any age group [15]. The cause of AD is unknown, only a small number of cases (<1%) have known genetic causes [33]. Genetic studies have found linkage between familial AD and presenilin 1(PS-1; chromosome 1), presinilin 2 (PS-2; chromosome 14) and amyloid precursor protein gene (APP; chromosome 21) [89]. The elderly age is the most significant non-genetic risk factor and AD is rare below the age of 50 [89].

The deposition of extracellular amyloid plaques containing amyloid beta (Aβ)  peptide and formation of intraneuronal neurofibrillary tangles are classical histopathological hallmarks of AD [68]. These findings are pronounced in the hippocampus, entorhinal cortex, amygdala, and cerebral cortex [89]. Aggregation of soluble Aβ into oligomer and large fibrils in different region of brain parenchyma is considered to be a crucial event in AD onset [62]. Aβ protein consists of a sequence of 39-42 amino acids derived from the transmembrane region of the APP [84]. The most predominant sequences of Aβ peptides are Aβ_1-40_ and Aβ_1-42_ [75]. Aβ_1-42_ accumulates early in amyloid plaques and polymerizes into fibrils more rapidly than Aβ_1-40_ *in vitro* [13, 78]. Studies using transgenic mice expressing APP have provided significant insight regarding the contribution of Aβ plaques to the AD pathology. In APP transgenic mice plaques are associated with neuronal death, neuritic dystrophy, dendritic spine loss and abnormal axonal morphology [90]. These altered morphologies lead to disrupted synaptic transmission [132]. Neurofibrillary tangles are composed of microtubule associated protein tau, normally expressed in axons, which are abnormally hyperphosphorylated in AD and aggregate into abnormal filaments in the neuronal cell body [65]. The number of neurofibrillary tangles is related to the presence and degree of dementia in AD [8]. Mutations of PS-1, PS-2 and APP in AD enhanced the processing of amyloidogenic Aβ from APP [124].

In recent years, adenosine, a purine ribonucleoside has drawn the interest of researchers for its neuromodulatory and neuroprotective effects in several neurological diseases. This endogenous nucleoside is found in all cells including glia and neurons, and plays important roles in the regulation of synaptic transmission and neuronal excitability in the central nervous system [38]. Adenosine exerts its cellular function through four G-protein coupled receptors (A_1_, A_2A_, A_2B_ and A_3_) [56], regulates, integrates and fine-tunes neuronal activity and influences several important brain functions such as sleep and arousal, cognition and memory, neuronal damage and degeneration [48, 102, 113]. The predominant pharmacological effects of adenosine are mediated through A_1_ and A_2A_ receptors. Several studies have investigated the expression of A_1 _and A_2A_ receptors in AD [6, 74] and tested the effects of A_1 _and A_2A_ receptor agonists/antagonists in animal models of AD [43, 104, 110]. Findings of enhanced cognitive function [66] or protection against cognitive impairment by nonselective blockade of A_1_ and A_2A_ receptors [7], and the enhanced release of several neurotransmitters including acetylcholine by modulating these receptors, made these two receptors a prime target for the development of new therapies for AD. In adult brain, adenosine concentration mainly depends on the activity of astrocyte-base enzyme adenosine kinase, which phosphorylates adenosine to AMP is considered to be the principal metabolic enzyme for the regulation of extracellular adenosine level in the brain [18]. Alternative metabolic routes for adenosine in brain that involve either deamination of adenosine to inosine by adenosine deaminase or reversible hydrolysis of S-adenosylhomocystein (SAH) by SAH hydrolase constitute minor pathways for the regulation of adenosine [18]. The role of adenosine kinase on adenosine metabolism in AD has not been studied yet. 

Different spatial distribution of adenosine receptors within the brain and their opposing effects after stimulation allow adenosine to exert a complicated effect for modulating other neurotransmitters [39]. The role of adenosine in different neurological diseases including epilepsy, stroke, chronic pain, Parkinson’s, Huntington’s, and Alzheimer’s disease has been elaborated briefly in review [17] based on their successful experimentation or clinical trial or therapeutic potential. This review attempts to summarize the role of adenosine reported in different studies and its diverse effects and association with AD.

## A_1 _RECEPTORS IN ALZHEIMER’S DISEASE

Adenosine depresses synaptic transmission and the release of various neurotransmitters acting through A_1_ receptors [58, 77]. In the hippopcampus, adenosine inhibits the release of acetylcholine and the excitatory amino acid glutamate [27, 50, 58]. Glutamate and its receptors have been implicated in the pathogenesis of AD and the dysfunction of this excitatory amino acid system may be responsible for some of the clinical manifestations of AD [32, 63]. Alexandre de Mendoca first described that endogenous adenosine, through A_1_ receptor activation, modulates long-term synaptic plasticity phenomena, such as long-term potentiation (LTP) [45], and subsequently showed that the tonic activation of A_1_ receptors decreases LTP, long-term depression and depotentiation [46, 47]. In accordance with the notion that synaptic plasticity is the basis for learning and memory in different brain areas [16], adenosine correspondingly modulates rodent performances in various learning and memory paradigms [48]. Administration of adenosine receptor agonists (mainly A_1_) disrupts learning and memory in rodents [29, 67,  97] while the nonselective adenosine receptor blockade by caffeine/theophylline or selective blockade of A_1_ and A_2A_ receptors improve the performances of rodent different behavioral tasks [67, 88, 100]. 

A_1_ receptors are highly enriched in the CA1 region of hippocampus [53, 79] in a normal healthy brain. A change in the pattern of A_1_ receptor expression has been found in AD patients when compared with age-matched control brains [6]. Most of the studies carried out in AD models for adenosine receptors were performed in hippocampus and striatum and have shown reduced levels of A_1_ receptors in these areas [74]. In AD patients, a reduced density of A_1_ receptors along with reduced binding sites for adenosine agonists and antagonists has been found in the molecular layer of the dentate gyrus. In addition, altered binding of adenosine agonists and antagonists to A_1_ receptors in CA1 and CA3 regions of hippocampus has been observed [139]. Kalaria and colleagues have demonstrated that A_1_ receptors are significantly reduced by 40-60% in AD after assessing the hippocampal samples collected from postmortem AD subjects. They also observed the highest reduction of A_1_ receptors in the molecular layer of the dentate gyrus including perforant pathways [82], which is the principal source of cortical input to the hippocampal formation [73]. However, little was known about A_1_ receptors in the frontal cortex of AD until a very recent study reported by Albasanz *et al*. [2]. The authors have shown that upregulation of A_1_ and A_2A_ receptors take place in frontal cortex both in early and advanced stages of AD, associated with sensitization of the corresponding transduction pathways. In agreement with theses results, a study carried-out in a transgenic mouse model (APP Swedish mutation) also found the higher levels of cortical A_1_ and hippocampal A_2A_ receptors as compared with the non-transgenic mouse [7]. The loss of A_1_ receptors appears to be among the factors responsible for cell death in CA1 [98, 117].

It is not clear whether A1 receptors influence the processes involved in the formation of abnormal APP and the formation of hyperphosphorylated tau in AD patients. However, the role of A1 receptors in APP processing, tau phosphorylation and cellular signaling has been studied in a model using human neural cells (neuroblastoma SH-SY5Y cells) that naturally express A1 receptors [6]. In this study, activation of A1 receptors led to the production of soluble APP, which was confirmed by the use of the A1-selective antagonist DPCPX. A1 receptors mediate tau phosphorylation and its translocation towards the cytoskeleton of neuroblastoma cells. A marked increase in A1 receptor immunoreactivity has been found in degenerating neurons with neurofibrillary tangles and in dystrophic neurites of Aβ plaques in the hippocampus and frontal cortex of AD. A significant co-localization of A1 receptors and Aβ in senile plaques and of A1 receptors and tau in neurons with tau deposition has been found [6]. The positive involvement of A1 receptors in in vitro APP processing and in tau phosphorylation and the presence of A1 receptors in the neurodegenerative structures of AD suggest that A1 receptors may play a role in the pathogenesis of AD. 

## A_2A _RECEPTORS IN ALZHEIMER’S DISEASE

A_2A _receptors have low expression in healthy brain but this pattern of expression and functionality can be changed in pathological conditions. Limited data is available about the distribution of A_2A _receptors in AD. An increased expression of A_2A _receptors in microglial cells in the hippocampus and cerebral cortex of AD patients has been found [6]. Long-term noxious stimuli to the brain increase the density and efficiency of A_2A_ receptors, which has been observed in animal models of Parkinson’s disease [101]. Some other studies also depicted the up-regulation of A_2A_ receptors in chronic stressful condition, which prevail in neurodegenerative diseases, with simultaneous decrease in the density of cortical A_1_ receptors [108]. Apart from AD, adenosine A_2A_ receptors have also been detected in the cerebral cortex of other neurodegenerative diseases such as Pick’s disease and a marked increase in A_2A_ receptors has been detected in the frontal cortex in Pick’s disease [3]. 

Modulation of A_2A _receptors could have neuroprotective effects in AD, in a way that it might interfere the pathogenesis of AD and increase the resistance of neuronal cells to insults. The prevailing hypothesis for progressive neurodegeneration that occurs in AD is the neurotoxicity caused by Aβ [144] and current evidence favors the idea that soluble Aβ plays the pivotal role in the pathogenesis of AD. Primary cultures of cerebellar granule cells with Aβ 25-35 in the presence of adenosine receptor blockers revealed that the blockade of A_2A_ receptors almost completely prevented Aβ induced neurotoxicity [43]. So it appears that the presence of A_2A_ receptor is essential for Aβ toxicity and inactivation of this receptor might limit the Aβ induced neurotoxicity in AD. The mechanism of neuroprotection by A_2A_ receptor antagonists against Aβ induced neurotoxicity is not known. One plausible hypothesis might be its ability to modulate neuro-inflammation by its anti-inflammatory properties [61].

Symptomatic benefit from cognitive impairment in AD patients might be achieved by modulating A_2A _receptors as these receptors could facilitate the neurophysiological mechanisms of learning and memory. Several studies revealed the potential modulatory role of brain A_2A _receptors on learning, memory and other cognitive functions [36, 121]. Delayed memory deficits induced by intracerebroventricular Aβ were prevented by either caffeine or selective A_2A _receptor antagonists [42]. This result indicates that caffeine affords its beneficial effects through A_2A _receptors, which were found to be up-regulated in cortical regions both in animal models [7] as well as in cortical tissues of patients with AD [2, 6]. The data collected from a recent report demonstrated that pharmacological blockade or genetic inactivation of A_2A _receptors has been found to attenuate memory loss induced by Aβ [36]. In A_2A_ receptor knockout mice, administration of Aβ did not cause learning deficits or synaptotoxicity [34], and this highlights the importance of the A_2A_ receptor in cognitive function. Aβ–induced cognitive disruption may be a result from synaptic dysfunction [25], so effective modulation of synaptic location might be helpful to counteract this cognitive impairment. The A_2A_ receptor antagonist ZM 241385 enhanced social memory in healthy rats [103] and reversed social memory impairment in spontaneously hypertensive rats [105] and this type of social memory is dependent upon the integrity of hippocampus [11]. The A_2A _receptor antagonist SCH 58261, improved the performance of mice in an inhibitory avoidance task in a time and dose dependent manner [88]. A_2A_ receptor antagonists usually confer the beneficial effects that have been found in different behavioral studies *in vivo*.

In contrast with cognitive benefits found by antagonizing the A_2A_ receptors, beneficial effects have also been reported by stimulating the A_2A _receptors in *in vitro* studies. Activation of A_2A _receptors by exogenous selective agonists facilitates the acetylcholine secretion and enhances glutamatergic synaptic transmission in hippocampus, but it is interesting to note that activation of these receptors by endogenous adenosine has no influence in basal hippocampal synaptic transmission [37, 80, 127]. Enhancing effects of A_2A_ receptor agonists on synaptic transmission has been found more prominent in aged animals [110] and this effect is most likely due to the increased expression of A_2A _receptor in the limbic cortex of aged rats [93]. Brain-derived neurotrophic factor (BDNF, a member of the neurotrophin family) and its specific tyrosine kinase receptor (TrKB) are highly expressed in the hippocampus [87] and BDNF has an important role over synaptic plasticity in adult hippocampus [95]. BDNF enhances LTP both *in vivo* [96] and *in vitro* [83] and this facilitatory action of BDNF upon LTP is completely lost upon blockade of A_2A_ receptors and depletion of extracellular adenosine [55]. Neurotrophins are potent survival factors for developing and degenerative neurons [147]. A decrease in levels and/or reduced activity of neurotrophins has been implicated in the pathophysiological mechanism of many neurological diseases including AD [122]. So the use of these naturally occurring neurotrophic factors poses promising therapeutic potential for the treatment of these neurological disorders [122]. The neurotrophins cannot cross the blood brain barrier so an invasive approach is required for *in vivo* administration. Neurotrophins can use transmembrane tyrosine kinases, so an alternate pathway is to use transactivation pathway such as A_2A_ receptor agonists, which can activate TrKB independent of neurotrophin binding [91, 147]. This A_2A_ receptor induced facilitation of neurotrophins raises the interest to use A_2A_ receptor agonists as a tool that can readily cross the blood brain barrier to potentiate neurotrophic actions in the brain. Despite all these beneficial effects, A_2A_ receptor induced excitotoxic glutamate mediated neuronal damage limits the option of A_2A_ receptor agonists as a therapeutic tool. Moreover, A_2A_ receptors become desensitized even after short-term exposure to agonists [126] and A_2A_ receptor agonist mediated desensitization of A_1 _receptors [99] made the A_2A_ receptor agonism less favorable for therapeutic potential. Although these *in vitro* stimulatory effects of A_2A_ receptors are interesting, but generalizability of these effects to *in vivo* situation has its limitations and further research is warranted in this regard. 

## CAFFEINE AND ALZHEIMER’S DISEASE

Caffeine, the most widely consumed behavioral stimulant present primarily in coffee and tea acts most likely by increasing cortical activity, cerebral energy metabolism and extracellular acetylcholine concentration [93]. Increased state of alertness and arousal is experienced due to caffeine-induced CNS stimulation [57]. Acetylcholine might be involved in caffeine’s stimulant properties as cholinergic nerve terminals arising from forebrain cholinergic complex [125] have been involved in arousal and attentional processes [54, 119]. Caffeine is one of the major contributors to the dietary antioxidants, which prevent neurons, and other cells from free radical induced oxidative damage and thus reduce the risk of chronic degenerative diseases [134]. It also augments the rate of cerebral glucose utilization [57], which could contribute to enhance cognitive function. Caffeine is a natural methylxanthine, a non-selective A_1 _and A_2A_ adenosine receptor antagonist, and a known neuromodulator with the associative effect on cognitive function, motor behavior and information processing [57]. Activation of A_1_ receptors inhibits excitatory synaptic transmission through presynaptic inhibition of glutamate release [137] and at the postsynaptic terminal it inhibits potassium conductance leading to neuronal hyperpolarization [64]. The excitatory effects of caffeine are likely to antagonize these receptors and this antagonism might explain the increased cognitive performances found with habitual caffeine consumption. The A_2A_ receptors are not so abundant or widely distributed in the brain like A_1_ receptors and caffeine block these excitatory A_2A_ receptors that has been reported neuroprotective consistently in several studies [14, 42, 43]. The A_2B_ and A_3_ receptors are not well characterized and the role of caffeine over these two receptors is not well documented. The A_2B_ receptors are found to be involved in the modulation of normal synaptic transmission and long-term potentiation in the CA1 region of the hippocampus [86], so this receptor might be of interest regarding AD related dementia. 

The effect of caffeine on cognition is an unresolved question and it is still debatable whether caffeine directly affects cognition [76]. There is a wealth of studies suggesting that the effects of caffeine on memory performance depend on the age [66, 76, 81, 114] and on the time of the day [10, 118] and sex [81, 114], but all these variables are often not adequately controlled or incorporated in most of the studies. The role of caffeine and selective antagonist of A_1_ and A_2A_ receptors on cognitive function has recently been extensively reviewed by Takahashi and colleagues [135]. In this review, it has also been stated that the effects of caffeine and of A_1_ and A_2A_ receptor antagonists primarily depends on the dose, the schedule and timing of administration, and on the mode of administration. Improved memory performances in rodents have been observed after moderate doses of caffeine ingestion [5, 31, 104], whereas higher doses of caffeine disrupt memory acquisition [4, 28]. The effects of caffeine on memory retrieval showed mixed results with some studies showing improvement [5, 4], while others have indicated either no response at all [59, 106] or even impairment in retrieval [28]. This disruption of acquisition or retrieval induced by caffeine might be due to the concomitant participation of both A_1_ and A_2A_ receptors with various effects of different brain regions that control the different phases of memory processing. The role of A_1 _and A_2A_ receptors in the control of synaptic plasticity is particularly important for caffeine in restoration of cognitive function when it is impaired. Inactivation of both A_1 _and A_2A_ receptors has been found to counteract the age related cognitive decline [104]. Because of these cognition enhancement properties from combined blockade of A_1 _and A_2A_ receptors, caffeine has been proposed as a potential therapeutic agent to reverse age-related cognitive decline [115].

A limited number of studies have investigated the relationship between coffee consumption and AD with regard to dementia or cognitive impairment.

### Experimental Animal Models

The potential neuroprotective benefits of caffeine consumption are reported from long-term caffeine intake rather than short-term ingestion that have been typically seen in experimental models. Administration of caffeine shortly before enhancing cerebral ischemia showed negative effects [133] while chronic administration of caffeine to gerbils for 4 weeks was able to reduce the neuronal damage in a model of global forebrain ischemia. The animal model does not spontaneously develop age-related changes that resemble AD pathology. To model AD in rodents, the first transgenic mice model that represents several histopathological features of AD was the PDAPP mice [60] and more recently, transgenic mice that overexpress APP with high level of brain Aβ  with aging have been developed [140]. Long-term caffeine administration in experimental AD (APP_SW_) transgenic mouse has demonstrated significant improvement in multiple cognitive tasks compared to that of control mice [7]. Reduced levels of soluble Aβ fragments have been found in these same transgenic mice and these effects are probably mediated through reduced expression in PS-1 and the β–secretase gene [7]. Caffeine also reduced the production of Aβ_1–40_ and Aβ_1–42_  peptides in neuronal cell cultures from these same transgenic mice [7]. Caffeine prevented neuronal damage and cognitive deficit caused by Aβ, an effect mimicked by A_2A_ receptor antagonists [42, 43]. These results further strengthen the idea that caffeine affords its beneficial effects on memory performance through its action on A_2A_ receptors.

An elegant study carried out by Herzog *et al*. has postulated that there is a link between a hypofunctioning cholinergic system and age-related cognitive deficits and has shown that the medial prefrontal cortex of aged rats is less liable to release acetylcholine upon depolarization [71]. An inverse relationship between adenosinergic and cholinergic systems has been reported in different studies where acetylcholine release tends to reduce in aged brains while adenosine content is increased [35, 131]. Since adenosine can attenuate the release of several neurotransmitters including acetylcholine [30, 41], it might be plausible to postulate that adenosine accumulation plays an important role in age-related cognitive deficits and hence may be considered an interesting target for pharmacological manipulation. Caffeine has an influence on acetylcholine and its receptors in the brain whether it is administered for a short time or a prolonged period. Administration of caffeine (both short-and long-term) to rats showed increased acetylcholine concentration in the prefrontal cortex without development of any tolerance that has been found in dopamine response [1]. The stimulatory effects of caffeine on acetylcholine release are probably mediated through A_1 _receptors because blockade of A_2A_ receptors by KF 17837 does not affect the acetylcholine release from rat cortical slices [19]. Moreover, the A_1_ receptor antagonist DPCPX has been found to stimulate cortical acetylcholine transmission [1], which is in agreement with *in vitro* [19] and *in vivo* [21] studies showing that A_1 _receptors are involved at multiple sites of action in regulating cholinergic neuronal activity. Long-term ingestion of caffeine by mice exhibited the increased number of muscarinic and nicotinic receptors in the brain and may also have increased cholinergic activity [128]. This augmented cholinergic function induced by caffeine would greatly benefit AD, where the cholinergic function disrupts with progression of the disease.

Finally, a possible mechanism underlying the association of caffeine consumption and cognitive functioning can be drawn from animal experiments. After consumption, caffeine enters the blood stream and acts as an A_2A_ receptor antagonist in the brain [57] which stimulates the cholinergic neurotransmitters which in turn prevents Aβ–induced neurotoxicity, a precursor of cognitive decline [43]. 

### Human Studies

A limited number of epidemiological studies have been conducted to determine the role of caffeine in neuroprotection and cognitive function in elderly and AD patients. The majority of epidemiological studies are retrospective case-control studies that compare the risk factors for AD present in AD cases with nondemented controls [141]. Although there is no consensus from the result, several studies have indicated improved cognition after long-term caffeine consumption. The effects of caffeine on cognition have consistently been highest among the oldest age group.

Several studies of short-term caffeine consumption have largely found that caffeine induces small but significant improvements in vigilance and psychomotor performances rather than improvements of higher-function such as memory and information processing [57, 111]. Scopolamine, a cholinergic blocker is used to induce cognitive deficits in healthy volunteers that mimic the cognitive deficits seen in AD. Short-term exposure of caffeine has been shown to improve memory and cognitive function in the presence of scopolamine induced impairments [116]. This result suggests that caffeine has specific memory enhancing properties and that the cholinergic pathway is involved in this process.

In contrast to the findings presented in short-term studies, multiple studies have shown that long-term (years to decades) caffeine consumption may result in improved cognitive function or may reduce the decline of cognition and memory that are found in AD and the aging process. Most of the longitudinal studies have consistently found the cognitive benefits of long-term caffeine consumption among the group of oldest individuals. 

A case-control study performed in a relatively small number of subjects compared the daily intake of caffeine in AD patients with non-demented matched controls. The patients with AD had an average daily caffeine intake of 73.9 ± 97.9 mg during the 20 years that preceded diagnosis of AD whereas the controls had an average intake of 198.7 ± 135.7 mg during the corresponding 20 years. The main finding of this study was a significant inverse association between caffeine intake and AD, and this association was independent of other causative factors that might influence caffeine intake and potential risk factors for AD [94]. 

In a prospective study known as the “Three City Study” examined the association between caffeine intakes, cognitive decline and dementia incident among the subjects aged 65 years and more. This 4-year long population based study concluded without finding any relation between coffee consumption and dementia in men but psychostimulant properties of caffeine appear to reduce cognitive decline in women without dementia especially at higher ages [114].

A 10-year prospective cohort study (the FINE study) in 676 men in Finland, Italy and the Netherlands found that the coffee consumption reduces cognitive decline in elderly men. Their findings suggest an inverse and J-shaped association between the numbers of cups of coffee consumed and cognitive decline, with the least cognitive decline for men consuming three cups of coffee per day [143]. 

The Canadian Study of Health and Aging (CSHA), is a longitudinal study of dementia in elderly people that mainly focused on its prevalence, incidence and risk factors. Caffeine was one of the factors among other risk factors; although this study was not specifically designed to assess the link of caffeine intake to AD. In this study, the daily coffee drinking has reduced the risk of AD by 31% among the age group of ≥65 years old during a 5-year follow-up [92]. The Manitoba Study of Health and Aging is a parallel study to the CSHA study, which found no influence of daily caffeine consumption during 5 years in AD incidence after adjustment for age, sex, and education [138]. 

The most recent population-based study after an average follow-up of 21 years found that coffee drinkers at midlife had lower risk of dementia and AD at later life, compared with that of non-drinkers or little drinkers. The risk of dementia reduced about 65% in people who drank 3-5 cups of coffee per day [52].

Cross-sectional studies reported contradictory findings with some found cognitive benefit [66, 76] while others depicted inverse relation between coffee consumption and AD [20]. Habitual caffeine consumption may improve cognitive function in the elderly and it is widely believed that it might have a protective effect in AD [76, 81]. There are some studies denoting an insignificant or no role at all for caffeine consumption on cognition and AD [138, 142]. 

This series of findings presented above indicates that caffeine and adenosine receptor antagonists affect memory performance both in humans and in animal models. 

## TREATMENT OPTIONS

The patients with AD are treated clinically principally with anticholinestarase inhibitor and N-methyl D-aspartate receptor antagonists, have been shown to slow-down the cognitive decline slightly. Antioxidant vitamin C and vitamin E [51] also showed slight benefit and all these available treatments are only confined in symptomatic interventions and they do not prevent neuronal degeneration and death. Several important therapeutic strategies either in clinical trial or in preclinical stage are briefly summarized below. 1) Immunization against Aβ either active or passive is currently a widely adopted experimental approach which already been translated into human clinical trial [123]. The aim behind this approach is to reduce the amyloid burden and enhance the clearing by redistributing the cerebral peptide to the systemic circulation [44]. Active immunization of APP transgenic mice provided highly desirable protection without any adverse effect influenced for safe clinical trail in mild to moderate AD patient, which unfortunately halted because a small fraction of these patients experienced aseptic meningoencephalitis. Further improvement for this vaccination therapy is underway to avoid the autoimmunity and proinflammatory reactions. 2) Inhibition of either of the two proteases β and γ–secretase, that produce Aβ from APP is another target researchers are trying to explore. Immunization of mouse model with Memapsin 2, a β–secretase, reduces the level of Aβ [22]. 3) It has been observed that some nonsteroidal anti-inflammatory drugs (NSAIDs) exerts beneficial effects by reducing neurotoxicity in the brain and decrease the level of selective Aβ42 without inhibiting the typical cyclooxygenase pathway [145], probably acting *via* allosteric modulation of γ–secretase. In a recent clinical trail NSAIDs administered to patients with mild cognitive impairment did not show any difference in the progression of AD between NSAIDs and placebo [120, 136]. 4) Metal chelation has been proposed as a therapy of AD after finding association of metals with β–pleated sheets of Aβ42 [67]. APP transgenic mice treated with antibiotic Clioquinol, a well-known Cu^2+^/Zn^2+^ chelator has been found impeding the deposition of Aβ and this strategy is at the verge of clinical trial stage [24, 72]. 5) Increased level of cholesterol is associated with a higher risk of cognitive impairment and dementia and connection between serum cholesterol level and risk of AD is suggested in several reports [107]. In preclinical studies high cholesterol-fed rabbit model exhibited increased accumulation of Aβ in brain and cholesterol-lowering drugs have been shown to reduce pathology in transgenic mice [112, 130]. Cholesterol lowering drugs statins are already well prescribed, widely used and tolerated drugs, so any trial based on this drug will be well suited for human and which are in way for clinical trial. The current pharmacotherapy of AD mainly focused on two aspects, 1) to increase the availability of acetylcholine in the central cholinergic system and 2) to ameliorate behavioral disturbances. The cholinergic hypothesis is supported by many studies, demonstrating that dysfunctional cholinergic system is sufficient to create memory deficit in animal models that are analogous to AD dementia [12]. Moreover, degeneration of the cholinergic neurons of the basal forebrain has been found in the brain of AD patients [9]. 

Due to profound neuroprotective and neuromodulatory effects of adenosine [40], adenosine receptors in the brain have drawn high research interest and are suggested as a potential target for therapeutic interventions in neurodegenerative diseases. Caffeine and A_2A_ receptor antagonists may contribute to AD pharmacotherapy by 1) augmenting cholinergic pathway and thus improving the cognitive functions and 2) by reducing brain Aβ burden and thus reducing the Aβ-induced neurotoxicity. It is well documented that the cognitive protection by caffeine involves the blockade of A_1_ receptors in hippocampus and cerebral cortex. Blockade of A_1_ receptors on cholinergic terminals has been shown to increase extracellular levels of acetylcholine [1], a neurotransmitter particularly important for cognitive processing that dramatically decreased in AD brain. In AD, the A_2A_ receptor is of high interest regarding therapeutic potential. A_2A_ receptor might provide symptomatic benefit and cognitive enhancement to AD patients. Disturbances in declarative memory are usually the earliest and most prominent clinical symptoms associated with AD and the most affected brain regions are hippocampus and related anatomical structures, as well as basal forebrain nucleus that provide cholinergic innervation to the hippocampus and neocortex [109]. Increased density of A_2A_ receptor in hippocampus of AD patients [6] and their location to presynaptic nerve terminals in hippocampus made them a suitable target for controlling neurotransmitter release and synaptic function [109]. Improved performance found in different behavioral tasks in animal studies is usually mediated by blocking A_2A_ receptors. Consistent cognitive improvement found in different longitudinal studies after long-term caffeine consumption indicate that caffeine plays important role to prevent cognitive decline in AD. A recent significant finding from an *in vitro* study gives a promising breakthrough that caffeine could attenuate Aβ burden [7] and presence of A_2A_ receptor is necessary for Aβ-induced neurotoxicity [43]. Therapeutics that reduces brain Aβ levels could provide cognitive benefit to AD that has been justified by improved cognitive function found in AD transgenic mice [26]. Enhanced cognitive function observed from nonselective blockade of A_2A_ receptor by caffeine or by selective blockade of A_2A_ receptors, made this receptor antagonist mechanism more favorable while exploring AD treatment. Caffeine is quite potent, non-toxic and as effective as selective A_2A_ receptor antagonists and the selective A_2A_ antagonist has not proven superior to caffeine neuroprotection [23, 43]. It would be premature to recommend that therapeutic use of caffeine would halt or reduce the cognitive decline in AD before performing rigorous pre-clinical and clinical trials on its therapeutic potential. But based upon the existing data collected from epidemiological studies of human caffeine consumption and from animal experimentation that pharmacological agents that mimic caffeine and selective A_2A_ receptor antagonists provides the rationale to evaluate their therapeutic potentials in the treatment of AD.

## CONCLUSION

In more than two decades since the adenosine receptors were cloned, quite a large number of studies reported from many groups throughout the world highlight the role of adenosine in different systems of the body. The role of adenosine in brain function has received most attention due to its different protective and modulating effects to CNS. The role of endogenous adenosine in preventing epileptic seizure is now a well-established fact drawn from different successful studies [146]. Selective adenosine A_2A_ receptor antagonists are currently under clinical trials for treating Parkinson’s disease with encouraging results [85]. Different therapeutic approaches have been contemplated for treating AD, the most common and devastating neurodegenerative disease of human intellect. Cognitive functions are badly affected and gradual deterioration is the inevitable outcome of this disease. Long-term effect of caffeine in AD patients seems to be most compelling among other proposed therapies for AD. The paradoxical neuroprotective effects from both activation and inactivation of A_2A _receptors reflect the complexity of actions of A_2A _receptors in CNS. Clear understanding of the cellular basis for neuroprotection afforded by A_2A _receptor antagonist would help to improve the design of clinical trials for AD. Time- and site-specific modulation of A_2A _receptors along with disease progression would benefit while formulating the adenosine-based therapy. Interpreting the data from epidemiological studies and animal models, it indicates that our understanding of the role of adenosinergic system in dementia is still far from complete. Complex interaction and plasticity between A_1_ and A_2A _receptors and their differential expression in different regions of the brain make it difficult to find the individual role and explicity of these two receptors. To achieve better understanding, integration between different types and phases of memory as well as differential expression of receptors in different brain area need to be evaluated. Combined neuroprotective findings of caffeine on cognitive function and AD incidence in both experimental and human studies are quite promising. To answer some conflicting reports, it is important to standardize the methodology of caffeine consumption measurement and relate the cognitive capacity with standard AD criteria.
